# Raman Thermometry of Confined Gas Milli-Flows

**DOI:** 10.3390/s26196344

**Published:** 2026-10-08

**Authors:** Guzmán Tejeda, Carlos Álvarez, José M. Fernández

**Affiliations:** Laboratory of Molecular Fluid Dynamics, Instituto de Estructura de la Materia IEM-CSIC, Serrano 121, 28006 Madrid, Spain; g.tejeda@csic.es (G.T.); carlos.alvarez@csic.es (C.Á.)

**Keywords:** Raman spectroscopy, temperature mapping, confined gas flow, millimetric channel, nitrogen, carbon dioxide, slip regime

## Abstract

The temperature of rarefied gas flows inside small channels is hard to measure without disturbing the flow. We present here an optical approach with submillimeter spatial resolution, based on Raman spectroscopy, and several examples of its application to gas flows within millimeter-sized channels. For this work, we have designed and built several channels with different geometries, and forced thermal gradients between some of their surfaces while flowing N_2_, CO_2_ and H_2_ through the channels. The pressures ranged from atmospheric down to 11 mbar, and the flow velocities ranged from 8 to over 450 cm/s. The most rarefied flows lie in the slip regime (Kn ≥0.001). Raman spectra of the flowing gases were recorded inside the channels, and the intensities of the rotational lines were analyzed in terms of Boltzmann plots to retrieve the local gas temperature. It is demonstrated that Raman spectroscopy is able to measure the temperature profiles across dimensions as short as 1 mm inside the channels. The achieved precisions (1-σ uncertainties) at 100 mbar are 0.8 K for CO_2_, 2.0 K for N_2_, and 3.7 K for H_2_.

## 1. Introduction

Currently, microfluidic devices with internal gas flows are the subject of rapid development and their applications span several scientific fields, from aerospace engineering to biology. Examples of such microsystems are micronozzles [1,2], micro-heat exchangers [3,4], micro-actuators [5], micro-thrusters [6,7], micro-gas chromatographs [8,9], gas sensors and separators [10,11,12], and vacuum micropumps [13], to name a few. Due to the low pressures and small dimensions of the systems, the Knudsen number Kn=λ/L, where λ is the molecular mean free path and *L* is a characteristic dimension of the channel, becomes non-negligible, implying that these devices often operate in moderately rarefied gas flow regimes [14,15]. In those rarefied flows, non-equilibrium effects and discontinuity of some flow quantities are expected to occur, like velocity slip and temperature jump near the channel walls. Furthermore, the optimum performance of those devices depends critically on the heat transfer and temperature distribution inside the gas flow. However, there is a lack of experimental techniques capable of measuring the internal properties, like the temperature, in gas flows confined in small channels [15], where conventional techniques are too intrusive or cannot be applied at all.

In this context, Raman spectroscopy is a promising optical tool based on the inelastic scattering of light [16]. Raman spectroscopy has become a powerful non-intrusive technique to probe unconfined gas flows at the molecular level with a spatial resolution of a few microns. These capabilities have been widely demonstrated in gas free microjets of small molecules at the Laboratory of Molecular Fluid Dynamics of the IEM-CSIC [17,18,19,20,21].

The main advantages of Raman spectroscopy for the present purpose are: (i) universality, in the sense that all gases composed of molecules give rise to the Raman spectrum; (ii) high spatial resolution of a few microns; and (iii) wide spectral range (>4000 cm^−1^). The main limitation is the intrinsic weakness of the Raman effect, along with the fact that the Raman signal can be obscured by fluorescence or phosphorescence from windows or from the sample itself. Thus, a highly sensitive instrument, with good rejection of the dominant elastic scattering is often needed for the practical realization in gaseous samples.

Raman spectroscopy relies on the structure of the Quantum-Mechanical energy levels of the gas molecules, like other molecular spectroscopies based on the absorption or emission of microwave or infrared radiation. Therefore, it can access the population of the energy levels of the molecular internal motions (rotation and vibration). At local thermal equilibrium, the populations obey the Boltzmann distribution [22] and the local temperature of the gas can be accurately retrieved from the Boltzmann plot [23].

Previous applications of Raman thermometry to confined gas flows are scarce. Walter et al. [24] and later Newton et al. [25] were able to measure the temperature of N_2_ inside optical pumping cells by means of its rotational Raman spectrum. More recently, Koschnick et al. [26,27] reported 1D and 2D Raman temperature profiles inside catalytic flow channels, with submillimeter resolution. However, the dimensions of the channels were of the order of centimeters in all those previous works.

The main goal of this paper is to evaluate the diagnostics capabilities of Raman spectroscopy to gas flows confined within millimeter-sized channels. We present here several examples of the application of Raman thermometry to such confined gas flows. For this work, we have designed several channels with different geometries, and forced thermal gradients between some of their surfaces while flowing gases through the channels. The pressures ranged from atmospheric down to 11 mbar, and the flow velocities ranged from 8 to >400 cm/s. The most rarefied flows lie in the slip regime (Kn >0.001).

The experiments carried out so far have demonstrated that the gas temperature in the flow can be mapped with high spatial resolution, from the rotational and vibrational Raman spectra of molecules like N_2_, CO_2_ and H_2_.

## 2. Materials and Methods

A graphical scheme of the experimental setup is shown in Figure 1. The Raman scattering was excited with 0.5–3.0 W at 532 nm from a Nd:YVO_4_ cw laser, focused onto the gas flow inside the channels by means of a f=35 mm lens, and collected at 90° with respect to both the laser propagation and gas flow directions. The laser polarization at the scattering volume could be rotated between *z* (parallel) and *y* (crossed) directions by means of a half wave plate. The Raman scattering was collected by means of a f=50 mm, *f*/1.8, photographic lens (Nikon, Tokyo, Japan ) and an achromatic doublet, and projected onto the entrance slit of the spectrometer with a total ×10 magnification. The latter was a Jobin-Yvon U1000 (Horiba, Lyon, France) 1 m dispersive double monochromator, equipped with 2400 grooves/mm gratings and a high sensitivity CCD detector cooled at −120 °C by liquid nitrogen [19]. The entrance slit was set to between 50 and 200μm, with the former yielding a spectral resolution better than 0.3 cm^−1^, able to fully resolve the rotational lines of gaseous CO_2_. The collection optics project a true image of the gas flow field on the entrance slit, and later on the detector surface. Thus, the dimensions *a* and *b* of the sampled volume (Figure 1, inset) can be controlled by the width of the entrance slit and the binning of the CCD detector. Typical values were a=5–15 μm, b=180 or 540 μm, and c=15 μm.

We designed and built four channels with rectangular sections and forced thermal gradients between some of their surfaces, with gases flowing through the channels. Channel C1 was 3D-printed by stereolithography (SLA) using black resin. It has an internal section of 1×5 mm^2^ and a total length of 20 cm, with two ISO-KF16 fittings at its ends. The gas was heated at the inlet, and a thermal gradient was created along the flow direction (*z* in Figure 1). The channel has apertures with BK7 optical windows for probing the gas flow at z=5, 10, and 15 cm downstream from the gas inlet. The apertures consist of two orthogonal pairs of large and small windows crossing the channel section. A photograph of the finished channel C1 after gluing the windows is shown in Figure 2a. The small windows (5 mm diameter), connected to the main channel section through a narrow passage (1 mm diameter), were used to pass the exciting laser, while the larger ones (10 mm diameter) were used to collect the Raman scattering from the gas flow inside the channel.

Channels C2a, C2b and C2c share a “sandwich” configuration made of two metal plates embracing a separator. Channel C2a was made of Al/PEEK/Al, and channel C2b was made of Al/inox/Al, both with ∅6 mm tube and standard Swagelok fittings, while channel C2c was made of brass/inox/brass with ISO-KF16 fittings. A photograph of channel C2a is shown in Figure 2b. Channel C2a has an internal section of 1×5 mm^2^ and a total length of 120 mm, while channels C2b and C2c have 1×15 mm^2^ and total lengths of 120 and 140 mm, respectively. The design and materials of the C2a-C2b-C2c sequence were bounded by the low thermal conductivity separators within the manufacturing possibilities, and their evolution was driven by improvements in the gas inlet/outlet geometry and in the leak tightness for working with rarefied gas flows. The external metal plates have two sapphire optical windows with a clear aperture of ∅15 mm. When the exciting laser hits one window at 45°, the Raman scattering at 90° can be collected through the same window, as indicated by the green and red arrows in Figure 2b. One of the metal plates was heated by an electric resistor, while the opposite plate was “cooled” by forced air convection from the fan in Figure 2b. This way, a thermal gradient was created along the short 1 mm dimension of the channel section (*y* in Figure 1).

The channels were mounted on translation stages, which could be moved along three XYZ orthogonal directions by micrometer screws, for optical alignment and precise control of the sampled gas volume within the channels. Channel C1 was mounted on an additional long-range micropositioner along *Z* to select the window to probe. The channels’ inlet and exit were connected through flexible tubing to gas feeding and exhaust lines, respectively. The gas feeding lines were provided with Bronkhorst mass flow controllers for accurate and constant flow rates, and the exhaust line was connected to a vacuum pump through an Equilibar back pressure regulator or a needle valve for accurate and constant sub-atmospheric pressures inside the channels.

## 3. Results

The experimental conditions for the gas flows studied in this work are summarized in Table 1. Flow velocities *v* (cm/s) were estimated as

(1) $$v=\frac{\dot{m}}{60}\;\frac{1013}{p}\;\frac{T}{273}\;\frac{1}{A}$$

where $\dot{m}$ is the mass flow rate in SCCM, *p* is the pressure in mbar, *T* is the absolute temperature in K, and *A* is the channel section in cm^2^. The Knudsen numbers were estimated as

(2) $$Kn=\frac{k_{B}T}{\sqrt{2}p\sigma L}$$

where $k_{B}$ is Boltzmann’s constant, σ is the collision cross-section of the gas, and L=1 mm is the shortest dimension of the channel. The collision cross-sections employed were 0.52, 0.43, and 0.27 nm^2^ for CO_2_, N_2_ and H_2_, respectively [28]. Reynolds numbers ranged from 0.1 to 480, and thus all the experiments were run under laminar flow conditions.

In all cases, the gas temperatures were determined from the spectral intensity of the rotational lines, measured as their integrated area in the Raman spectra. The pure rotational S(J) lines were used for N_2_ and CO_2_, while for H_2_, the rotational Q(J) lines of its vibrational band at ∼4160 cm^−1^ were used instead [28]. The rotational Raman spectra of N_2_ and CO_2_ were recorded under crossed polarization (*y* in Figure 1) to minimize the Rayleigh (elastic) scattering, while the highly polarized vibrational Q branch of H_2_ were recorded under parallel polarization (*z* in Figure 1). An example of a rotational Raman spectrum of CO_2_ inside channel C1 is shown in Figure 3. Each of the lines in the comb-like structure is due to a J→(J+2) transition between rotational energy levels of CO_2_. It must be remarked that the Rayleigh scattering at 0 cm^−1^ and the stray light from the channel walls and windows was blocked very efficiently by our spectrometer, allowing us to see close-lying lines like the S(4) at 8.6 cm^−1^ with good quality.

The Raman intensity $I_{J}$ of the rotational lines of a linear molecule is given by

(3) $$I_{J}=C\;{\left(\nu_{0}-\Delta \nu \right)}^{4}\;P_{J}\;F\left(J\right)$$

where *C* is a coefficient depending on molecular and geometrical properties (which is constant for a given experiment); $P_{J}$ is the normalized ($\sum_{J}P_{J}=1$) population of the initial energy level *J* of the transition, i.e., the fraction of molecules in that energy level; and F(J) is the line strength of the transition. For a Boltzmann distribution

(4) $$P_{J}\propto w_{J}\;\left(2J+1\right)\exp\left(-E_{J}/\left(k_{B}T_{R}\right)\right),$$

where $w_{J}$ is the nuclear spin statistical weight [28], $E_{J}$ is the rotational energy of the *J* level, and $T_{R}$ is the rotational temperature. For the J→(J+2) S(J) rotational lines of N_2_ or CO_2_

(5) $$F\left(J\right)=\frac{\left(J+1)(J+2\right)}{\left(2J+1)(2J+3\right)},$$

while for the J→J Q(J) rotational lines of the vibrational band of H_2_ the F(J) line strengths are listed in Table 2.

By plotting the following function of the rotational Raman intensities

(6) $$\ln\frac{I_{J}}{{\left(\nu_{0}-\Delta \nu_{J}\right)}^{4}\;w_{J}\;\left(2J+1\right)\;F\left(J\right)}=A-\frac{E_{J}}{k_{B}T_{R}}$$

against the rotational energies $E_{J}$ (so-called Boltzmann plot), a straight line is obtained, from whose slope the rotational temperature $T_{R}$ can be determined with great accuracy. The Boltzmann plot corresponding to the spectrum of Figure 3 is shown as an inset.

The results of the experiments carried out with the four channels will be discussed next.

### 3.1. Channel C1

Channel C1 was probed with N_2_ and CO_2_ gases. Before entering the channel, the gases passed through a reservoir (heat exchanger) at 100 C ($T_{1}$ in Table 1), and then through the ISO-KF16 inlet at a measured external temperature $T_{2}$. Rotational Raman spectra of N_2_ or CO_2_ were recorded under crossed polarization from the gas flow at the center of windows 1 and 2, at 5 and 10 cm downstream from the channel inlet. For each measured point, typically five independent spectra were recorded and averaged to check for repeatability.

The temperatures retrieved as explained above at the two windows of channel C1 for N_2_ and CO_2_ flows are plotted in Figure 4 as a function of the flow rate. The depicted error bars are 1-σ from the standard deviation of the linear fits to the Boltzmann plots. It can be seen that both the gas temperature at the two probed windows, and the difference between them, increase with the flow rate for the two gases studied in this work. The temperature difference between the two windows at increasing distances downstream from the inlet is an indication of the thermalization of the gas stream to the channel walls. This can be rationalized in terms of the contact time between the hot gas stream and the cold channel walls: the faster the flow, the shorter the contact time between gas and walls.

### 3.2. Channels C2a, 2b and 2c

Channel C2a was probed with N_2_ and a mixture of N_2_ and acetone vapor; channel C2b was probed with N_2_ and CO_2_ gases; and channel C2c was probed with N_2_, CO_2_ and H_2_ gases, as detailed in Table 1. The temperatures $T_{1}$ and $T_{2}$ in Table 1 are those of the hot and cold plates of the channel, respectively, as measured by external thermocouples.

By displacing the channels along the direction *y* of observation (red arrow in Figure 2b) the focus of the exciting laser (Figure 1, inset) probes the shortest dimension of the flow field (1 mm nominal), with a magnification of $\sqrt{2}$ due to the 45° geometry. The spatial resolution along the laser beam (*b* in Figure 1, inset) is determined by the height of the active area of the CCD, since the collection optical system projects a true image of the flow field on the 2D detector. When its full height is read, b=540 μm. However, this could be improved by splitting the CCD into smaller strips. This way, three shorter sections of b=180 μm were probed simultaneously.

The temperatures retrieved as explained above along the 1 mm dimension of the channels C2a, C2b and C2c are shown in Figure 5 for the flows #13–18 of N_2_, in Figure 6 for the flows #19–23 of CO_2_, and in Figure 7 for the flow #24 of H_2_. Again, the depicted error bars are 1-σ from the standard deviation of the linear fits to the Boltzmann plots. In all these figures, the hot side lies at the positive (right-hand) end of the horizontal scale. It must be remarked that the experiment of Figure 5b was run with a mixture of saturated acetone vapor and N_2_, demonstrating that the proposed technique can also be applied to gases in mixtures.

## 4. Discussion

Since all the flows were in laminar regime, the temperature profiles of Figure 5, Figure 6 and Figure 7 are the result of three competing physical processes: (i) the heat exchange between the gas and the channel walls; (ii) the heat conduction across the gas bulk; and (iii) the advection due to the flow velocity. The observed temperature profiles come from different experiments with different channels C2a, C2b and C2c, and thus they are not strictly comparable. In addition, CO_2_ has a strong absorption in the infrared, and thus can be heated by the thermal radiation from the cell walls, while N_2_ and H_2_ cannot. However, some general trends can be drawn from them.

At the same pressure, the precision of the temperature measurements is larger for CO_2_ than for N_2_, and the latter is larger than for H_2_. The best precisions achieved, estimated as medians of the 1-σ uncertainties, are as follows: for CO_2_, 0.8 K at p> 100 mbar and 2.7 K at 11 mbar; for N_2_, 1.5 K at 1 bar and 2.0 K at 100 mbar; and for H_2_, 3.7 K at 100 mbar. This is closely related to the number of observed rotational lines in each case, which translates into the number of points in the Bolztmann plot. On the other hand, for a given gas, measurements become noisier as the pressure is reduced, because the observed Raman intensity is proportional to the density of scatterers. This poses some actual limits as ∼10 mbar for CO_2_ and ∼100 mbar for N_2_ for practical realization of such measurements with our setup.

Interestingly, in many cases we were able to measure the temperature of the gas flow up to the walls of the optical windows. However, points close to the walls were certainly affected by luminescence from the window material, and are less accurate than those in the bulk of the flow. A representative example of the signal-to-noise ratio of the rotational Raman spectra of CO_2_ flow #23 along the channel width is shown in Figure 6f.

The gas pressure also has a larger effect on the temperature profile than the flow velocity, both for N_2_ and CO_2_. This can be concluded by comparing Figure 5d with Figure 5e, which show similar trends despite different *v*, and Figure 5c with Figure 5e, which show very different profiles despite similar *v*. The same conclusion can be reached by comparing Figure 6a–c for CO_2_. The N_2_ flows at pressures around 1 bar in Figure 5a–c show a more marked temperature gradient than those at 100 mbar in Figure 5d–f. This behavior can be rationalized in terms of the heat conduction: at larger density, the heat flux is hindered by the larger heat capacity of the gas volume, although the thermal conductivity coefficient is rather constant at the pressures of these experiments. All in all, this indicates that the flow velocity is too slow to compete with the transversal heat conduction across the 1 mm section of the flows.

The particular channel employed also has an effect on the temperature profiles. The flows in Figure 5f and Figure 6d,e, measured with the channel C2c, have a lower average *T* than those of the channels C2a and C2b. This can be due to a different efficiency of the gas-to-wall collisions for the aluminum (C2a and C2b) and brass (C2c) walls, or to the different temperature gradients achieved with the different channels: ΔT∼15 K for C2b, ΔT∼10 K for C2c. Furthermore, the different profiles between Figure 6b–d, with similar pressures, can also be attributed to the use of different channels.

It is worth discussing some possible limitations and sources of systematic errors in the current work. The first of them might be the risk of the flowing gas being heated by the exciting laser. None of the studied gases absorb at 532 nm, and thus the power transferred from the exciting laser into the gas volume is negligible. However, the laser can heat the inlet/outlet optical windows and these, in turn, heat to some extent the gas layer close to them. This effect would show up as a temperature increase close to both windows in the C2 channels, which is not observed in Figure 5, Figure 6 and Figure 7.

Second, all the temperatures reported in Figure 5, Figure 6 and Figure 7 are rotational temperatures $T_{R}$, which are expected to correspond to the translational or kinetic temperature $T_{K}$. However, in rarefied gas flows, the collision frequency decreases, and the measured $T_{R}$ can differ from $T_{K}$, as is the case in supersonic free jets. The collision frequency can be written in terms of the Knudsen number as

(7) $$f=\frac{2}{Kn\;L}\sqrt{\frac{2k_{B}T}{\pi m}}$$

where *m* is the molecular mass. For Kn=0.001, the degree of the most rarefied flows studied here, the mean free times τ=1/f range from 0.5 ns for H_2_ to 2.6 ns for CO_2_. By using the rotational collision numbers from [29], the corresponding rotational relaxation times range from 7 ns for CO_2_ to 0.2 μs for H_2_. These times are orders of magnitude smaller than the 0.01 s for the fastest gas flow to travel from the heating element to the observation window. Thus, for the gas flows studied in the present work $T_{R}$ can be expected to correspond to $T_{K}$, although this may not be the case in much more rarefied flows.

Third, vibration–rotation interactions can make the rotational matrix elements depart from the rigid rotor expression (5), jeopardizing the accuracy of the temperature determination from Equation (6). This effect is characterized by the so-called Herman–Wallis (HW) factors [30]. For the pure rotational Raman spectrum of N_2_, HW factors have been reported by Bohlin et al. [31], leading to a temperature decrease of 0.7 K at the temperatures of the experiments. In turn, the estimation of the HW factors for CO_2_ by Alvarez et al. [23] would lead to a decrease in the gas temperature of 0.7 K around room temperature. These deviations are smaller than other sources of uncertainty, and fall within the reported error bars. Furthermore, the previous works on Raman thermometry in confined flows [24,25,27] retrieved the gas temperature from Boltzmann plots using the line strengths in the rigid rotor approximation, like in the present work.

Finally, the rotational temperatures of H_2_ in Figure 7 have been determined considering that the 3:1 mixture of ortho-H_2_ and para-H_2_ relaxes as a single species, i.e., the populations of the J=0 to 4 rotational energy levels obey a common Boltzmann distribution. This assumption implies averaging the rotational temperatures of the ortho-H_2_ and para-H_2_ species. However, their difference is negligible at the temperatures of the present experiments and much smaller than the error bars in Figure 7.

## 5. Concluding Remarks

Raman spectroscopy is a non-intrusive technique that can measure local temperature in gas flows with high spatial resolution. Here, we demonstrated this capability in gas flows of small molecules (N_2_, CO_2_, H_2_) confined in millimeter-sized channels. The high sensitivity and long-term stability and repeatability of our setup allowed us to record useful Raman spectra down to 11 mbar of CO_2_. With a specially designed channel, we were able to probe the gas flow up to the channel walls. For the three gases studied in this work, the precision of the measured temperatures is maximum for CO_2_ and minimum for H_2_, with N_2_ in between. The measurements are more precise for higher pressures and points that are not too close to the windows walls. Typical/representative precisions at 1 bar are ±1 K for CO_2_, ±1.5 K for N_2_.

The experiments described in this work were run for hours. Since the minimum acquisition times for each individual spectrum were of the order of tens of seconds, this particular realization can be applied only to stationary flows, not to unstable or rapidly changing flows. The performance of the current setup can be improved in several ways. Notch optical filters can be used to block the stray light from the channel walls. Higher laser power and longer integration times can be used to increase the signal/noise ratio of the recorded Raman spectra. Finally, it would be of much interest to compare the results of the present work with numerical simulations of the confined flows to check for pitfalls and possible ways of improvement.

## Figures and Tables

**Figure 1:**
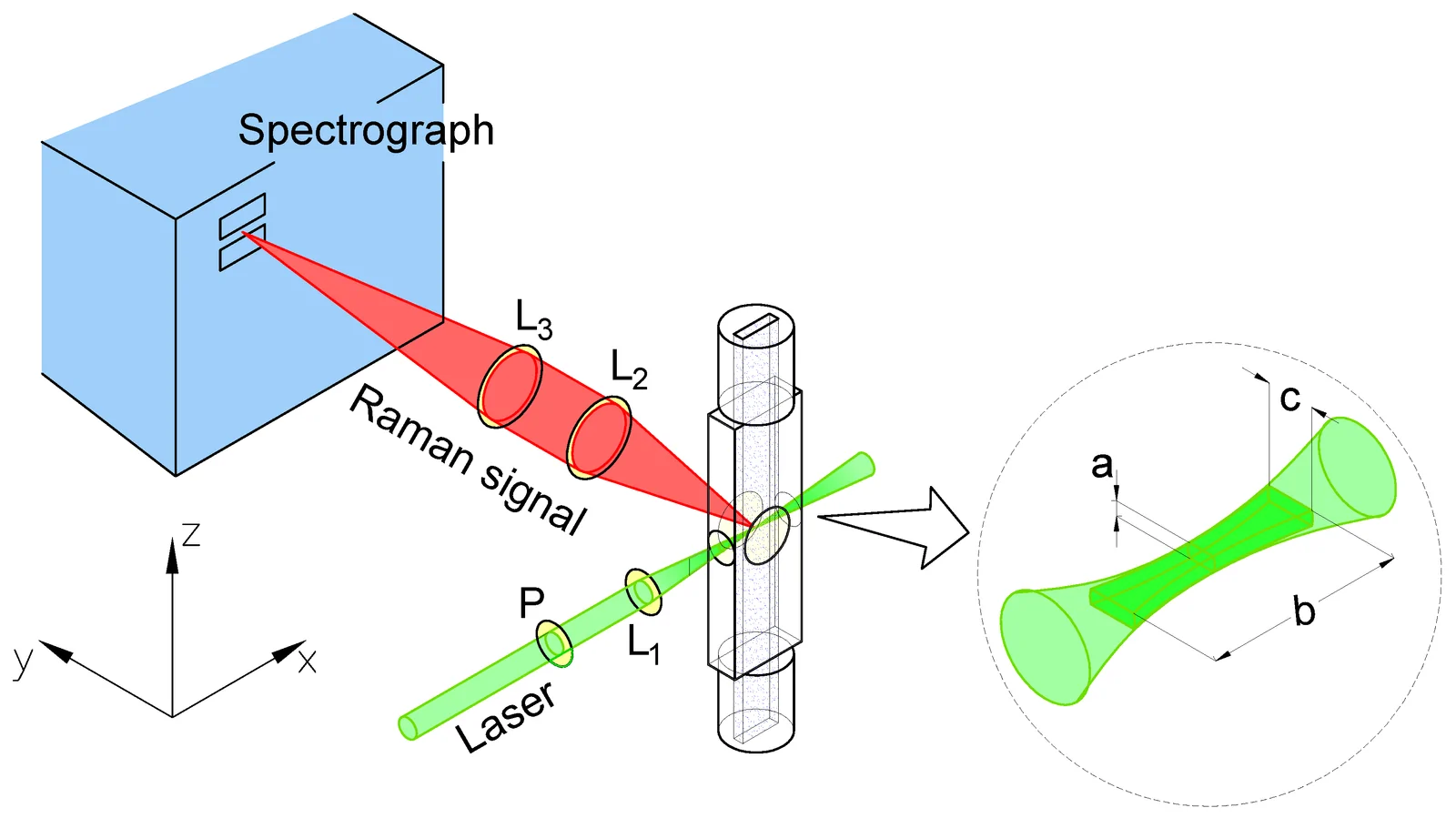
A scheme of the experimental setup for Raman thermometry of confined gas flows. The excitation laser beam, the Raman signal, and the gas flow propagate along *x*, *y* and *z* directions, respectively. P: half wave plate; L_1_: focusing lens; L_2_ photographic lens; L_3_ achromatic doublet. The inset shows a magnified view of the sampled volume; see the text for a, b, c dimensions.

**Figure 2:**
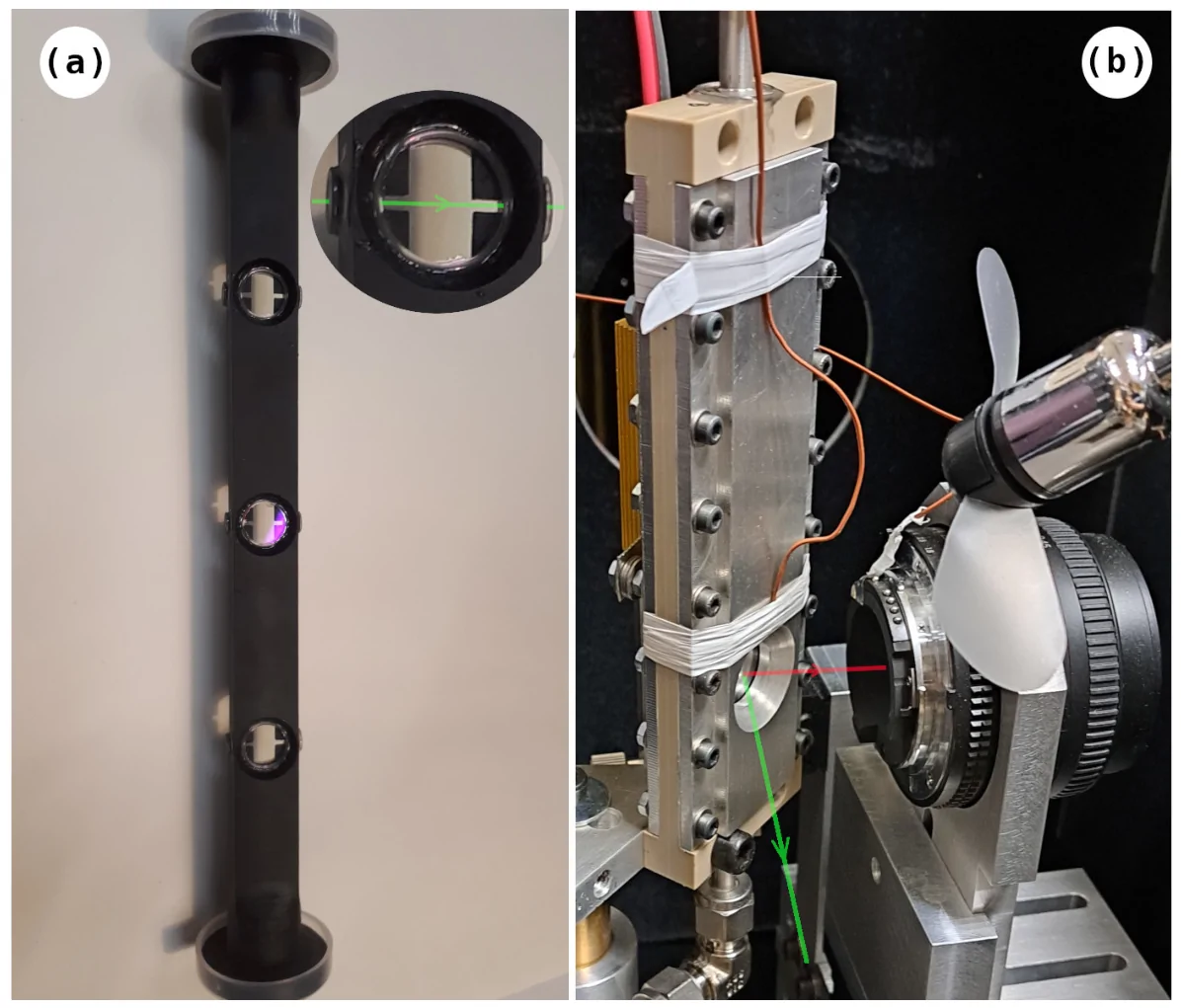
Photographs of some of the channels employed in this work. (**a**) Channel C1 made by 3D printing with glued windows, showing the 5 mm section through the observation apertures. (**b**) Channel C2a showing the two aluminum plates embracing the PEEK separator, and the Nikon photographic lens to collect the Raman scattering. The green arrows mark the direction of the exciting laser in both cases, and the red arrow in (**b**) marks the direction of observation.

**Figure 3:**
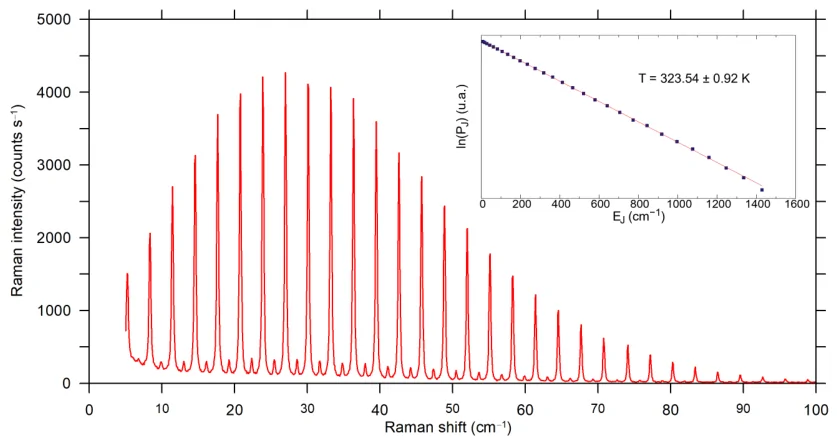
Rotational Raman spectrum of CO_2_ in flow #8 through channel C1. The inset shows the corresponding Boltzmann plot; $\ln(P_{J})$ denotes the LHS of Equation (6).

**Figure 4:**
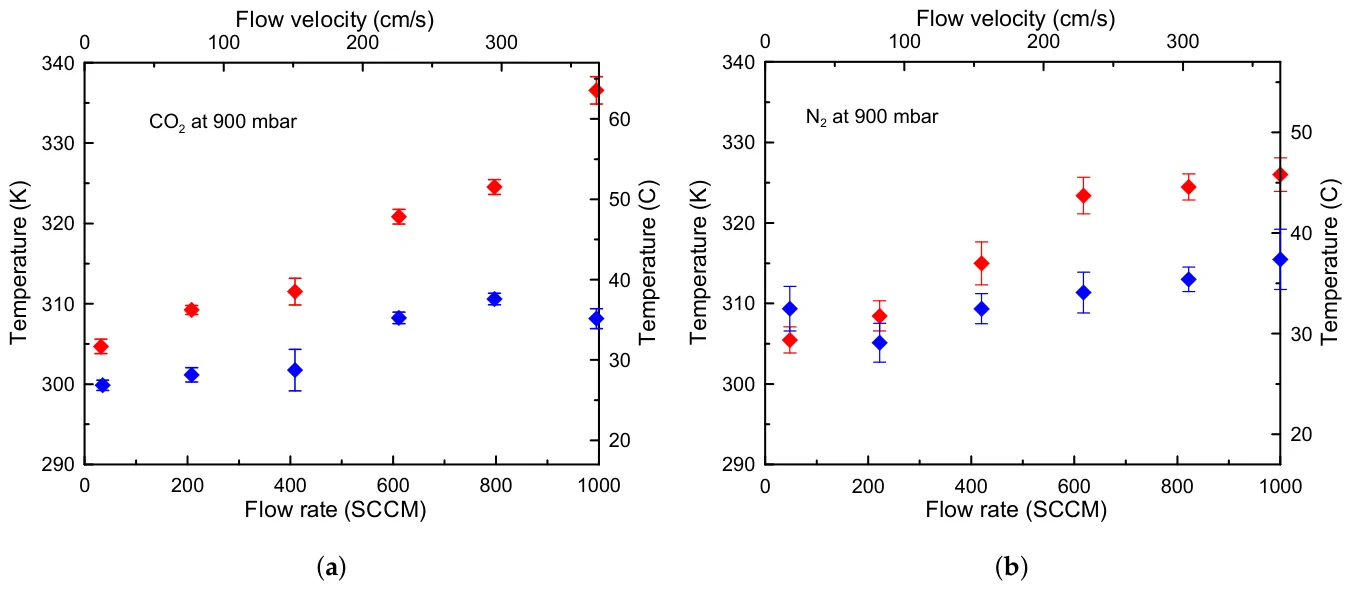
Rotational temperature of CO_2_ in flows #7–12 (**a**) and N_2_ in flows #1–6 (**b**) through channel C1, as measured at 5 cm (red symbols) and 10 cm (blue symbols) downstream from the channel inlet as a function of the flow rate.

**Figure 5:**
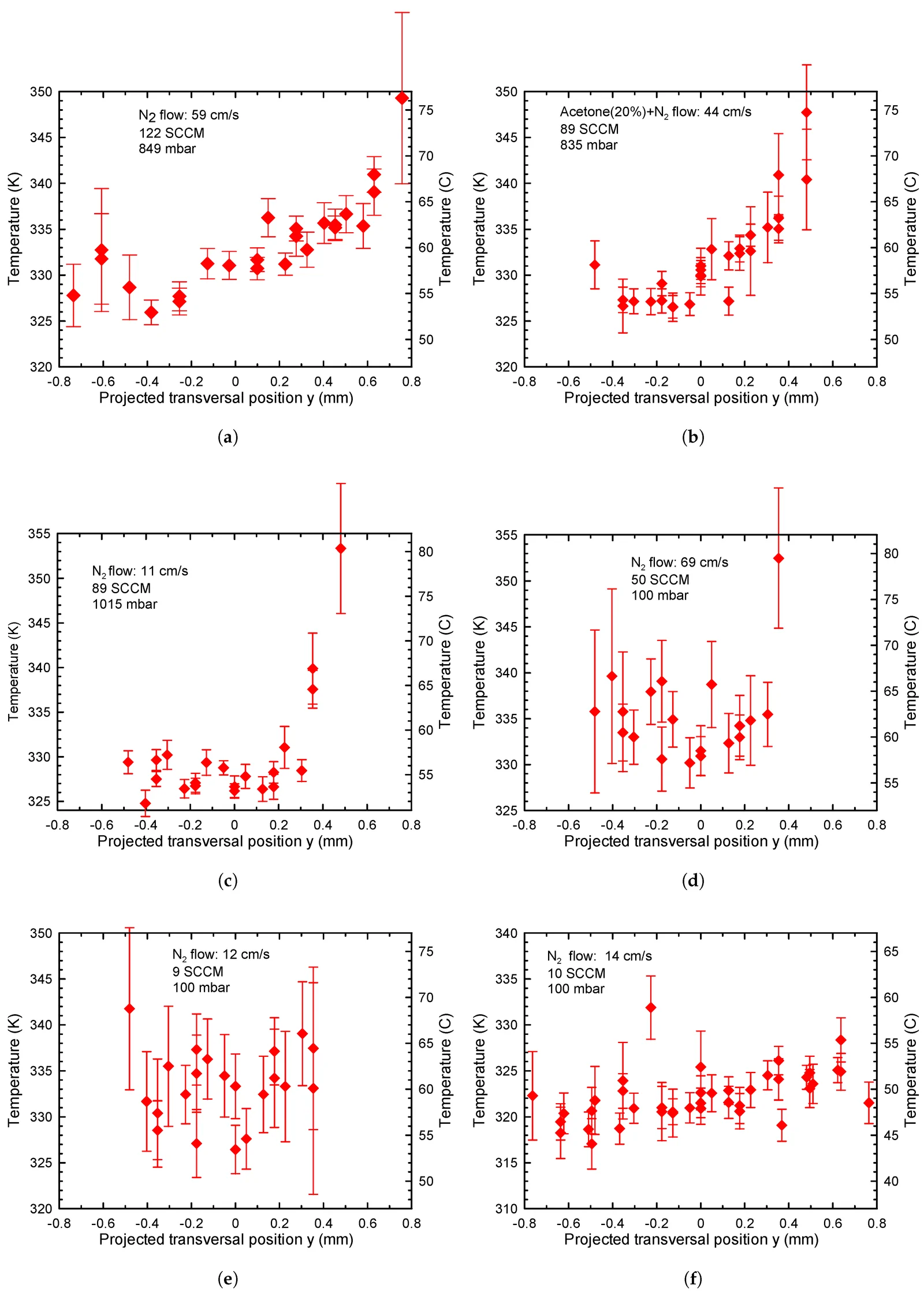
Rotational temperature of N_2_ in flows #13 (**a**), #14 (**b**), #15 (**c**), #16 (**d**), #17 (**e**) and #18 (**f**), as probed along the 1 mm section of channels C2a, C2b and C2c.

**Figure 6:**
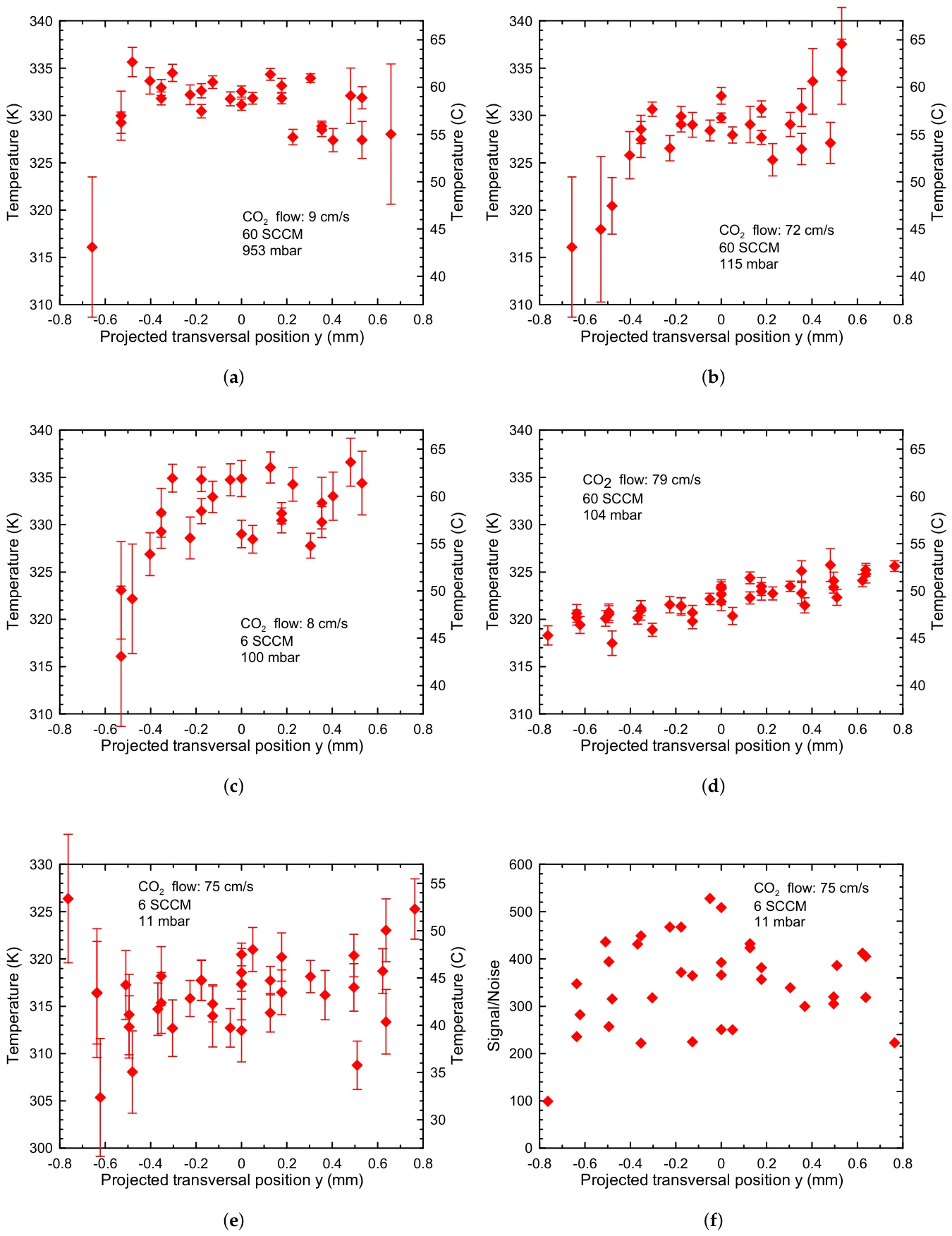
Rotational temperature of CO_2_ in flows #19 (**a**), #20 (**b**), #21 (**c**), #22 (**d**) and #23 (**e**), as probed along the 1 mm section of channels C2b and C2c; (**f**) signal-to-noise ratio of the rotational Raman spectra of flow #23.

**Figure 7:**
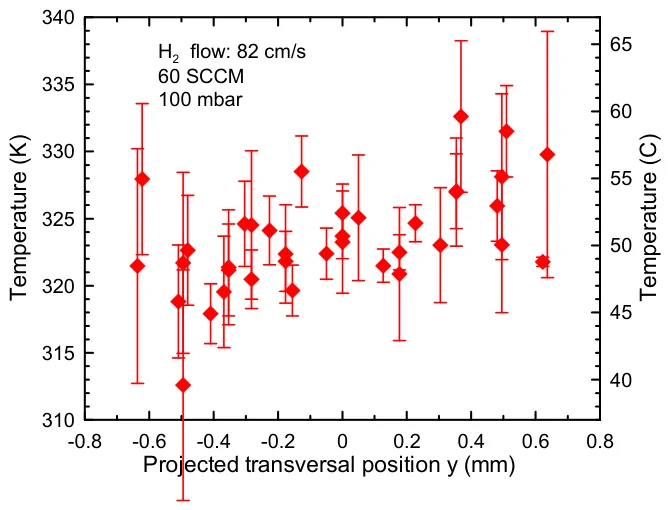
Rotational temperature of H_2_ flow #24, as probed along the 1 mm section of channel C2c.

**Table 1:** Experimental conditions for the gas flows studied in this work.

Flow #	Channel	Section (mm^2^)	Gas	Flow (SCCM)	*p* (mbar)	$T_{1}$ (C)	$T_{2}$ (C)	*v* (cm/s)	Kn
1	C1	1×5	N_2_	1000	906	100	55	448	8.2 × 10^−5^
2				822	901	100	55	370	8.3 × 10^−5^
3				618	904	100	55	277	8.2 × 10^−5^
4				420	896	100	55	190	8.3 × 10^−5^
5				222	906	100	55	99	8.2 × 10^−5^
6				48	890	100	55	22	8.4 × 10^−5^
7	C1	1×5	CO_2_	995	890	100	56	455	6.9 × 10^−5^
8				797	905	100	56	358	6.8 × 10^−5^
9				610	900	100	56	276	6.9 × 10^−5^
10				409	880	100	56	189	7.0 × 10^−5^
11				208	887	100	56	95	7.0 × 10^−5^
12				33	874	100	56	15	7.1 × 10^−5^
13	C2a *	1×5	N_2_	122	849	60	40	59	8.9 × 10^−5^
14			N_2_ + acetone	89	835	60	39	44	9.1 × 10^−5^
15	C2b *	1×15	N_2_	89	1015	60	44	11	7.1 × 10^−5^
16				50	100	60	44	69	7.6 × 10^−4^
17				9	100	60	44	12	7.6 × 10^−4^
18	C2c *	1×15	N_2_	10	100	60	50	14	7.6 × 10^−4^
19	C2b *	1×15	CO_2_	60	953	60	45	9	6.6 × 10^−5^
20				60	115	60	45	72	5.4 × 10^−4^
21				5.9	100	60	45	8	6.3 × 10^−4^
22	C2c *	1×15	CO_2_	60	104	60	48	79	6.0 × 10^−4^
23				6	11	60	48	75	5.7 × 10^−3^
24	C2c *	1×15	H_2_	60	100	60	50	82	1.2 × 10^−3^

* Channel C2a is Al/PEEK/Al, C2b is Al/inox/Al, and C2c is brass/inox/brass sandwiches.

**Table 2:** Line strengths F(J) for the Q(J) rotational lines of the vibrational band of H_2_ employed in this work *.

*J*	Δν (cm^−1^)	F(J)
0	4161.2	1.00
1	4155.2	1.03
2	4143.3	1.04
3	4125.8	1.06
4	4102.6	1.10

* Adapted from Equation (27) of [22].

## Data Availability

The dataset is available on request from the authors.

## Abbreviations

The following abbreviations are used in this manuscript:

CCD	Charge-coupled device
LHS	Left-hand side
MEMS	Micro-electro-mechanical system
PEEK	Polyether ether ketone
SCCM	Standard cubic centimeters per minute
